# Dezocine as preemptive analgesia alleviates ultrapulse CO_2_ fractional laser treatment induced pain in patients with acne scars

**DOI:** 10.1111/jocd.16410

**Published:** 2024-06-24

**Authors:** Qian Zhang, Xiaoqin Li, Yuanli Wang, Ni Gao, Rongli Zhang, Jingjing Wu, Fengqin Wu, Pu Song, Min Chen, Gang Wang, Lin Gao

**Affiliations:** ^1^ Department of Dermatology, Xijing Hospital Fourth Military Medical University Xi'an Shaanxi China; ^2^ Department of Anesthesiology, Xijing Hospital Fourth Military Medical University Xi'an Shaanxi China

**Keywords:** acne scars, dezocine, reduction of pain, ultrapluse CO_2_ fractional laser

## Abstract

**Background:**

Ultrapluse CO_2_ fractional laser technology has emerged as an effective treatment for scar management. However, one drawback of this modality is the pain caused during the procedure. This study aims to explore the efficacy and safety of dezocine (DZC) as preemptive analgesia for reduction of pain induced by ultrapulse CO_2_ fractional laser treatment for acne scars.

**Methods:**

The study cohort included 78 outpatients with acne scars between February and April 2023. Patients were randomly assigned into three groups with intravenous injection (iv) of DZC prior to laser treatment: (1) control, iv of saline; (2) DZC group 1 (DZC_1), iv of DZC at 0.15 mg/kg; and (3) DZC_2, iv of DZC at 0.20 mg/kg. After 30 min, one session of ultrapulse CO_2_ fractional laser treatment on acne scars was performed. Hemodynamics, visual analogue scale (VAS), and anxiety visual analog test (AVAT) were monitored prior to, during, and after the procedure.

**Results:**

Operative success rates for patients in the control, DZC_1, and DZC_2 groups were 34.6%, 84.6%, and 100%, respectively. DZC administered with either dosage significantly reduced the VAS and AVAT scores of patients in treatment groups as compared with the subjects in the control group during the course of ultrapulse CO_2_ fractional laser treatment. Patients in DZC_1 and DZC_2 groups did not show any significant difference in hemodynamic parameters, VAS, and AVAT scores. Temporary adverse effects such as nausea and dizziness were observed in some subjects after treatment; the symptoms were quickly dissolved after a rest in supine position.

**Conclusions:**

DZC as preemptive analgesia could effectively reduce pain and anxiety induced by ultrapulse CO_2_ fractional laser treatment in patients. This study provided an option of preemptive anesthesia to minimize the pain and discomforts associated with laser treatments in clinical practices.

## 1 INTRODUCTION

The ultrapulse carbon dioxide (CO_2_) fractional laser can remodel and rejuvenate skin textures through creating an array of micro treatment zones (MTZ) to promote cell proliferation and collagen production.^1^, ^2^ It has emerged as a highly effective modality for various skin conditions to improve facial wrinkles, elasticity, freckles, textures, photoaging spots, and scars.^3^, ^4^, ^5^, ^6^ Particularly, it has evolved as the main method for management of acne depressed scars.^7^, ^8^ However, adverse effects such as pain, anxiety, and discomforts induced by the CO_2_ fractional laser treatment are major drawbacks of this technology, which is ranked the third in order of importance to be addressed in one patient survey.^9^ Therefore, appropriate preemptive anesthesia will be necessary during ultrapulse CO_2_ fractional laser procedure to result in rapid sedation, leading to comforts in patients and enhancing physician efficacy.

Currently, there are several ways to alleviate photodynamic pain treatment, including surface anesthesia, local infiltration anesthesia, and general anesthesia, as well as auxiliary methods such as cooling ice packs, music relaxation, and psychological counseling.^10^ However, these methods all have their limitations: surface anesthesia can only relieve pain to a limited extent, local infiltration anesthesia has certain operational risks and many adverse reactions, and the most effective method is general anesthesia. However, due to the requirements for facilities, time, and costs of general anesthesia, it places a considerable burden on patients in terms of economy and psychology. In addition, considering the comprehensive after‐effects, there are certain obstacles to clinical application.

Dezocine (DZC) is a synthetic opioid‐like agent, introduced in 1970s as an analgesic and developed by Yangtze River Pharmaceutical Group in China in 2009.^11^ The mechanistic action of DZC to produce antinociceptive effects is thought through activation of κ and μ opioid receptors.^12^, ^13^ It also targets norepinephrine/serotonin transporter to inhibit uptake of norepinephrine and serotonin.^14^ DZC is considered as a partial agonist or a hybrid agonist–antagonist to opioid receptors, which renders advantages of low adverse effects (such as addiction and constipation), quick effect, and large safe‐dose range than those pure full agonists.^11^ Therefore, it may serve as a potential alternative to opioid anesthetic agents in response to the opioid overdose epidemic.^15^ For example, DZC has shown potential in the treatment of opioid dependence.^16^ DZC has been used as preemptive analgesia before many surgical procedures, such as abdominal operation surgery, laparoscopic cholecystectomy and gynecological laparoscopic surgery, endoscopic sinus surgery, and gastroscopy and colonoscopy procedures.^11^, ^17^ As preemptive analgesia, DZC has shown greater analgesic and sedative effects but lower adverse reactions than morphine. DZC has been combined with dexmedetomidine to reduce adverse reactions and inflammatory responses in patients undergoing hyperthermic intraperitoneal chemotherapy (HIPEC) after intestinal surgery.^18^ So far, DZC is the top 1 painkiller in China, accounting for 45% opioid analgesia used nationally.^11^ In this study, we attempted to explore the efficacy and safety of DZC as preemptive analgesia for reduction of pain induced by ultrapulse CO_2_ fractional laser treatment for acne scars.

## 2 METHODS

### 2.1 Study population

The study cohort included 78 patients with acne scars undergoing ultrapulse CO_2_ fractional laser treatment between February and April 2023. Risk factors and potential contraindications to using this treatment were fully discussed with patients before written informed consent was sought and obtained. The echelle d'evaluation clinique des cicatrices d'acne (ECCA) scale^19^ was used for acne scar. Patients with the following conditions or medical histories were excluded from this study: pregnancy, long‐term sedative use, hypertension (blood pressure over 160/100 mmHg), apnea, severe cardiovascular disorders, severe liver and kidney disorders, severe mental illness, communication disorder, trauma, acute and chronic alcoholism, hypotension, hypothyroidism, asthma, epilepsy, and allergy to DZC.

### 2.2 Treatment

All patients were given oral palonosetron (0.5 mg) to prevent gastrointestinal adverse reactions after the application of DZC and topical application of lidocaine cream (containing 25 mg of propylcaine and 25 mg of lidocaine per gram) on the scar areas 1 h before laser procedure. Patients were randomly assigned into three groups (*n* = 26 subjects per group) with intravenous injection (iv) of DZC 30 min prior to laser treatment: (1) control, iv of saline; (2) DZC group 1 (DZC_1), iv of DZC at 0.15 mg/kg; and (3) DZC_2, iv of DZC at 0.20 mg/kg. DZC was made into 10 mL of saline and administered within 30 seconds through iv Vital signs were monitored.

Patients were treated with ultrapulse CO_2_ fractional laser (UltraPulse® Encore; Lumenis, Santa Clara, CA, USA) with the Deep FX mode with 17.5–25 mJ, density 5, and 300 Hz. Additional treatment was followed using the Active FX mode with 70–125 mJ, density 3 and 75 Hz without overlapping throughout the entire face. A facial mask was applied after laser treatment.

### 2.3 Safety and efficacy evaluation

(1) Hemodynamic parameters including diastole and systole blood pressures (DBP, SBP), heart rate (HR), and oxygen saturation (SpO_2_) were monitored before DZC administration, the course of laser treatment, and 10 min post laser procedures. (2) Pain sensation from patients was assessed using visual analogue scale (VAS) with a scale from 0 to 10. (3) Anxiety visual analog test (AVAT) was used for anxiety evaluation with a range from 0 to 10, which was marked by patients. (4) Operative success rate was determined by the ratio of successfully completed operations to all attempted operations. An operation could be successfully completed if a patient showed a VAS score less than 4 and had active communications with physicians. If patients had a VAS score over 7 and couldn't endure the procedure, the operation would be stopped and failed. (5) Patient's self‐satisfaction with the procedure was recorded with categories from unsatisfied, neutral, satisfied, to very satisfied. (6) Anterograde amnesia was assessed at 30 min after laser treatment by asking memories of four events from patients: topical application of lidocaine cream, needle puncture of iv, the location of laser treatment started, and facial mask application. Loss of memories for all four events would be graded as full forgetfulness. Loss of one to three events would be scaled as partial forgetfulness.

### 2.4 Postoperative care and follow‐up

All patients were observed for any potential discomforts in our clinics for 3 h post laser treatment prior to being released to go home with a family member. A follow‐up call to patients or their family members within 24 h was made to check for and record any adverse effects.

### 2.5 Statistical analysis

The Graphpad 9.5 software was used for statistical analysis. $\bar{x}$ ±s and paired *t*‐test were used for quantitative analysis. *p* < 0.05 was considered statistically significant.

## 3 RESULTS

A total of 78 patients was enrolled in this study, including 43 female and 35 male subjects. The age ranged from 20 to 38 years old (28.69 ± 8.54). The skin types were Fitzpatrick III–IV. The average ECCA score was 72.24 ± 10.28. There were no differences in gender ratio, age, body mass index (BMI), and ECCA scores among the three groups (Table 1).

**TABLE 1:** Demographics characteristics of patients.

Group	Gender (M/F)	Age (years old)	BMI (kg/m^2^)	ECCA
Control	12/14	28.35 ± 9.73	23.31 ± 2.81	72.15 ± 10.25
DZC_1 (0.15 mg/kg)	11/15	29.12 ± 8.21	23.01 ± 2.74	72.54 ± 10.14
DZC_2 (0.20 mg/kg)	12/14	28.51 ± 9.31	23.25 ± 2.76	71.96 ± 10.46

The VAS and AVAT scores were recorded at four time points, for example, before treatment, at the beginning of the treatment, during treatment, and post treatment. Patients in both DZC treatment groups showed significant decreases in VAS scores at the beginning of the treatment and during treatment as compared with patients in the control group (Figure 1). Patients in both DZC treatment groups showed significant decreases in AVAT scores during treatment as compared with patients in the control group (Figure 1). There was no significant difference in VAS and AVAT scores between DZC_1 and DZC_2 groups (Figure 1). There was no significant difference in VAS and AVAT scores before and post treatments among all three groups (Figure 1).

**FIGURE 1:**
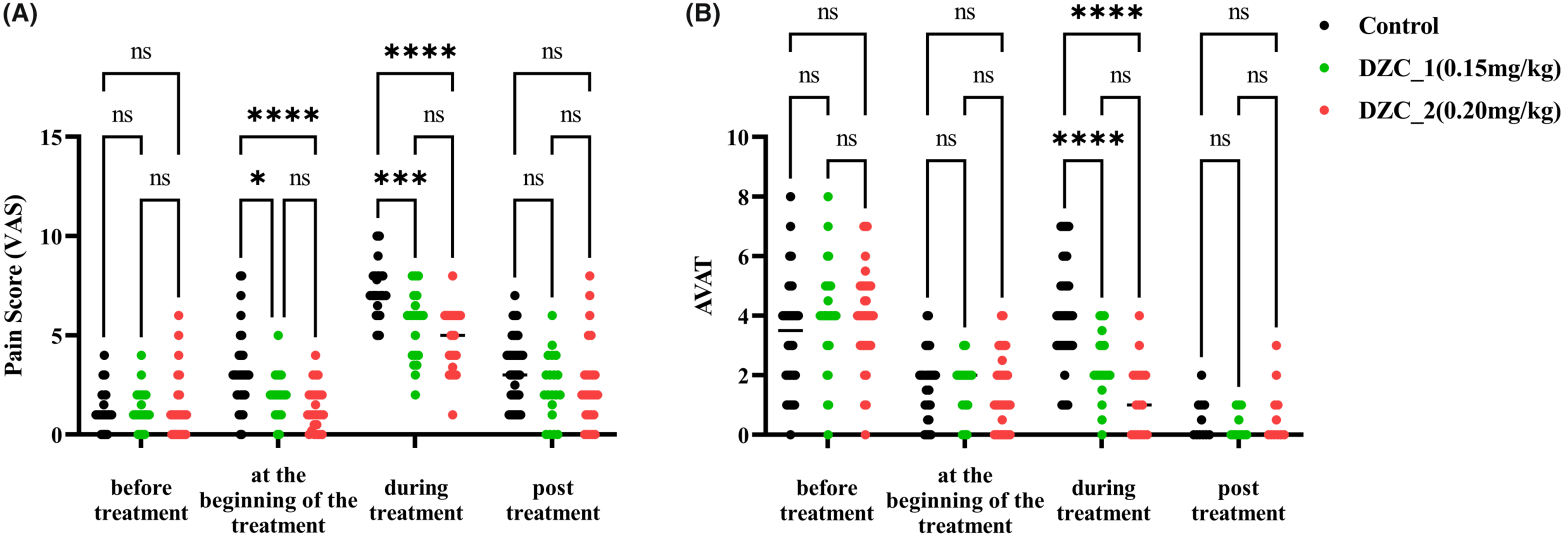
Comparison of VAS and AVAT scores among groups before, at the beginning of, during, and after laser treatment. A, both dosages of DZC significantly reduced VAS scores among patients at the beginning and during treatments as compared with the controls. B, AVAT score improvement was observed in both DZC treatment groups during the laser procedure as compared with the controls. *p < 0.05; ***p < 0.001; ****p < 0.0001; ns, no significance.

DZC significantly decreased HR during the treatment as compared to the control group (Figure 2). DZC treatments slightly lowered, but insignificantly, HR in patients as compared with the subjects in the control group after laser procedure (Figure 2). Patients in the DZC_2 group showed a significant increase in SpO_2_ during the treatment as compared with the patients in the control group (Figure 2). There was no significant difference in SBP and DBP before, during, and post treatments among all three groups (Figure 2).

**FIGURE 2:**
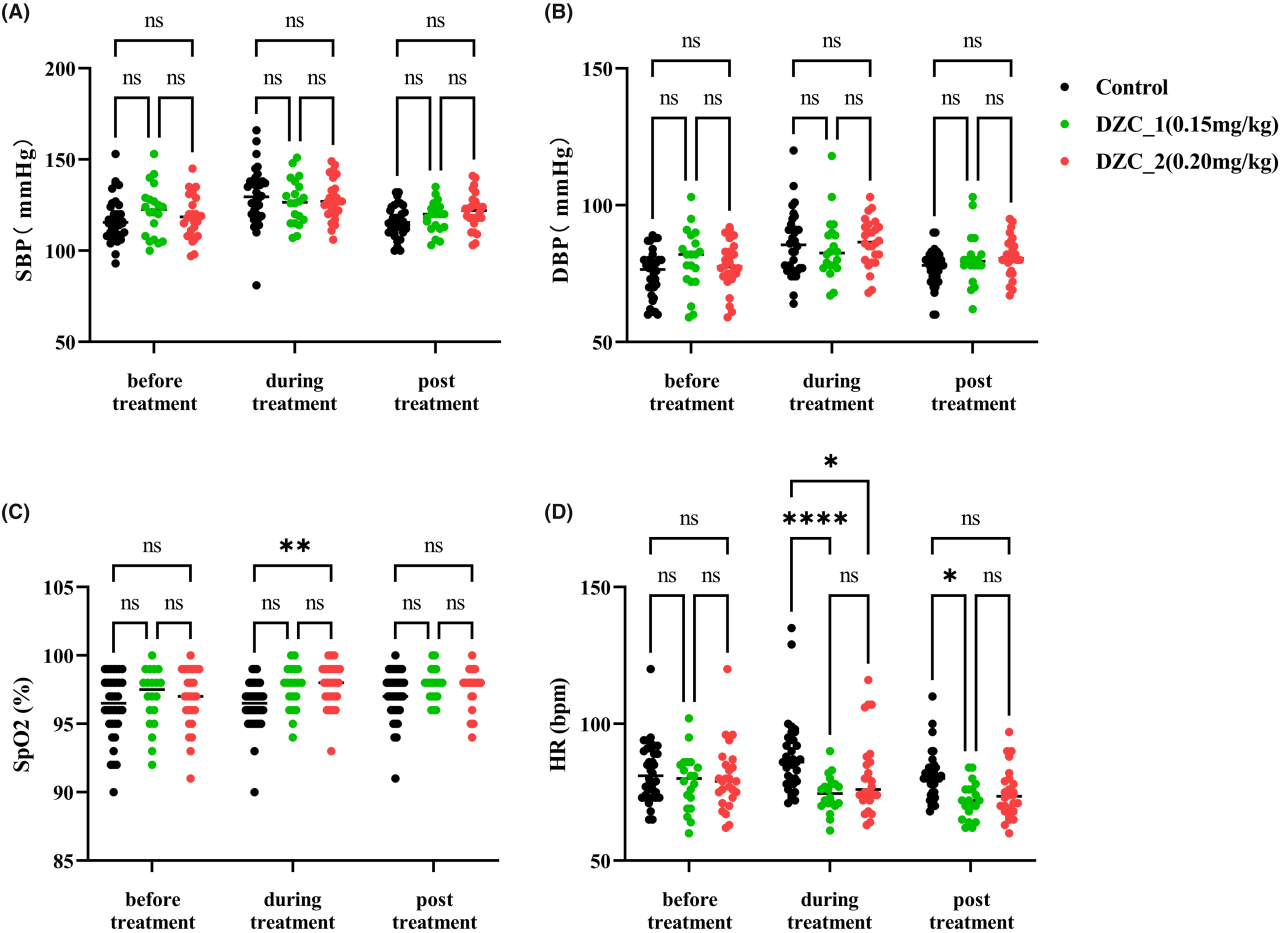
Hemodynamic parameters before, during, and post laser treatment. A and B, no significant difference was observed in SBP (A) and DBP (B) before, during, and post treatments among all three groups. (C) DZC with a dosage of 0.20 mg/kg significantly increased SpO_2_ in patients during the treatment as compared with the patients in the control group (*p* < 0.05). (D) HR was significantly decreased in patients in DZC_1 group (*p* < 0.0001) and DZC_2 group (*p* < 0.05) as compared with the patients in the control group during treatment. ns, no significance.

DZC substantially increased the laser operative success rate and patients' self‐satisfactions as compared to the control group (Table 2). Operative success rates for patients in the control, DZC_1, and DZC_2 groups were 34.6%, 84.6%, and 100%, respectively (Table 2). There was no anterograde amnesia observed among all groups (Table 2). Nausea and dizziness were observed within 3 h after laser procedures (Table 3). Those adverse events were temporary and quickly dissolved after a rest in supine position within 6–8 h. There were no side effects reported 24 h after laser treatment.

**TABLE 2:** Evaluation of operative success rate, patient self‐satisfaction, and anterograde amnesia among groups.

Group	Operative success rate	Self‐satisfaction				Anterograde amnesia
		Unsatisfied	Neutral	Satisfied	Very satisfied	
Control	9/26 (34.6%)	8 (30.8%)	9 (34.6%)	8 (30.8%)	1 (3.8%)	0/26
DZC_1 (0.15 mg/kg)	22/26 (84.6%)	3 (11.5%)	5 (19.2%)	15 (57.7%)	3 (11.5%)	0/26
DZC_2 (0.20 mg/kg)	26/26 (100%)	0 (0%)	6 (23.1%)	16 (61.5%)	4 (15.4%)	0/26

**TABLE 3:** Side effects associated with DZC administration.

Group	Nausea	Dizziness	Drowsiness	Respiratory suppression
Control	0	0	0	0
DZC_1 (0.15 mg/kg)	10	15	0	0
DZC_2 (0.20 mg/kg)	10	16	0	0

## 4 DISCUSSION

Opioids, such as morphine, oxycodone, hydrocodone, tramadol, carfentanil, and heroin, are commonly used for the management of acute and chronic pain. While these opioids provide potent analgesic effects, they also pose a high risk of addiction and can lead to respiratory depression and even fatal outcomes.^20^, ^21^ The misuse of these opioids has resulted in a significant and ongoing public health crisis known as the opioid epidemic.^22^

DZC is a novel opioid analgesic with similar efficacy to morphine. It is characterized by rapid onset of action, prolonged duration of effect, low potential for addiction, and minimal respiratory depression. Widely utilized in clinical settings, DZC not only serves as pre‐operative analgesia for various surgical procedures such as laparoscopic appendectomy, epidermal cyst resection, and thyroid surgery but also helps reduce postoperative agitation and alleviate pain in elderly patients undergoing general anesthesia.^11^, ^17^ Research indicates that intravenous administration of DZC at 0.1mg/kg reduces the incidence and severity of urinary catheter‐related bladder discomfort following open abdominal surgery compared to flurbiprofen exate at 1 mg/kg.^23^ Similarly, during maxillofacial surgery, the same dose of intravenous DZC significantly decreases postoperative sore throat while enhancing patient satisfaction.^24^ Additionally, it has been reported that DZC effectively alleviates physical withdrawal symptoms associated with morphine use and restores conditioned location preference behavior, suggesting its lower addictive potential and improved safety.^16^

In this study, we demonstrated the effectiveness of DZC as preemptive analgesia to sufficiently reduce pain and anxiety induced by ultrapulse CO_2_ fractional laser treatment in patients with acne scars. Our data also showed a good safety profile of DZC in a range of two dosages. This study provided an option of usage of DZC as preemptive anesthesia to minimize the pain and discomfort associated with laser treatments in clinical practices.

Ultrapulse CO_2_ fractional laser has shown its clinical efficacies in treatment of various skin conditions.^3^, ^4^, ^5^, ^6^ It has evolved as the front line of modality for acne scars.^7^, ^8^ Many studies have focused on the improvements of clinical efficacy of acne scar treatment using CO_2_ fractional lasers.^25^, ^26^ However, the pain and discomfort associated with CO_2_ fractional laser treatments are the major concerns to patients and physicians. Effective management of laser‐treatment‐associated pain in clinical practice should not be underrated. Anesthesia approaches for laser modalities can be categorized into noninvasive and invasive methods. Noninvasive methods include topical anesthesia and cryoanesthesia but ineffective anesthesia, systemic absorption, and allergic reactions are the potential drawbacks.^9^, ^10^ While ultrapulse CO_2_ fractional laser generally requires a moderate or relatively strong analgesic effect, thus invasive methods such as injected and supervised anesthesia could be considered. However, invasive anesthesia may result in an over analgesic level than CO_2_ fractional laser procedure needs. Furthermore, the invasive approaches are associated with many disadvantages, such as risks of adverse effects, needs of anesthetic expert and instrument for operation, and high costs. These are the barriers that hamper their applications in cosmetic laser settings for dermatologists. Therefore, preemptive analgesia could be an option for laser‐treatment patients that would render an appropriate level of analgesic effect with low risks and be readily available in most clinical settings.

In this study, we attempted to explore the preemptive analgesia strategy in our routine laser treatment procedures. DZC is considered as the most appropriate candidate since it offers several advantages. First, DZC is a hybrid agonist–antagonist to opioid receptors, resulting in lower risk of addiction and overdose than those pure full agonists.^11^ Second, the safety and efficacy profiles of DZC as preemptive analgesia have been established in many preoperative procedures.^11^, ^26^ Third, it shows quick action with low adverse effects and wide safe‐dose ranges.^11^ Fourth, it is available in most clinics and the cost is very low (~$15.00 per procedure), which is about 3% of the cost of general anesthesia procedure in China. In our study, patients in the DZC groups remained well conscious during the entire laser procedure while effective sedation was sustained to relieve the pain, leading to a high operative success rate. The hemodynamic parameters in patients remained relatively constant during the treatment, demonstrating that DZC had minimal effects on those vital signs. Temporary side effects such as nausea and dizziness were observed in many patients and dissolved quickly. Therefore, DZC provided an appropriate level of analgesic effect so that most patients showed well pain tolerance with few side effects and completed the laser procedures.

Beside, the entire study was conducted in compliance with research ethics and patient safety, with vital signs being monitored in a formal medical setting. Patients' blood pressure, heart rate, and partial oxygen pressure were measured before, during, and after treatment. No significant abnormal fluctuations were observed in the relevant indicators during the different monitoring periods, and all procedures and safety measures were carried out under the supervision of anesthesiologists.

## 5 CONCLUSIONS

Our data showed the efficacy of DZC as preemptive analgesia to manage pain and anxiety during ultrapulse CO_2_ fractional laser treatment in patients with acne scars. DZC also exhibited a good safety profile of few side effects and minimal effects on hemodynamics during the procedure. This study demonstrated that DZC could be implemented as preemptive anesthesia to reduce the pain and discomfort in routine laser treatment in clinics.

## AUTHOR CONTRIBUTIONS

All authors contributed to the study conception and design. Material preparation and data collection were performed by Qian Zhang, Lin Gao, Xiaoqin Li, Yuanli Wang, Ni Gao, Rongli Zhang, Jingjing Wu, Fengqin Wu. Data analysis were performed by Pu Song and Min Chen. The first draft of the manuscript was written by Qian Zhang and Lin Gao. Gang Wang and Lin Gao revised the manuscript. All authors read and approved the final manuscript.

## FUNDING INFORMATION

This work was supported by grants from the National Natural Science Foundation of China (82373499, 82173436).

## CONFLICT OF INTEREST STATEMENT

The authors declare no conflict of interest.

## ETHICS STATEMENT

The study was approved by the ethics committee at the Xijing Hospital (KY20202029‐F‐2), Clinical Trials.gov ID: NCT05861063.

## Data Availability

The data presented in this study are available on request from the corresponding author (Lin Gao, Email: gaolin@fmmu.edu.cn). The data are not publicly available due to privacy issues.
